# Recurrent angioedema, Guillain-Barré, and myelitis in a girl with systemic lupus erythematosus and CD59 deficiency syndrome

**DOI:** 10.1186/s13317-020-00132-2

**Published:** 2020-06-29

**Authors:** Vadood Javadi Parvaneh, Leila Ghasemi, Khosro Rahmani, Reza Shiari, Mahbobeh Mesdaghi, Zahra Chavoshzadeh, Seyed Hassan Tonekaboni

**Affiliations:** 1 Department of Pediatric Rheumatology, Mofid Children’s Hospital, Shahid Beheshti University of Medical Sciences, Shariati Ave, Tehran, Iran; 2 Department of Immunology and Allergy, Mofid Children’s Hospital, Shahid Beheshti University of Medical Sciences, Tehran, Iran; 3 Department of Pediatric Neurology, Mofid Children’s Hospital, Shahid Beheshti University of Medical Sciences, Tehran, Iran

## Abstract

**Background:**

CD59 deficiency is a congenital mutation disorder in complement pathway which can present with various manifestations.

**Case presentation:**

Herein, we presented an adolescent 16-years-old girl with recurrent attacks of Guillain-Barre in early childhood and then recurrent attacks of angioedema, paresthesia, and myelitis. Finally, she presented with quadriplegia, malar rash, proteinuria, lymphopenia, and high titer of antinuclear antibody. So, the patient developed systemic lupus erythematosus. Furthermore, we performed whole exome sequencing which revealed homozygote mutations in CD59 for the patient and heterozygote one for her parents. CD flow cytometry showed less than 1 percent expression of CD59 on the surface of the patient’s peripheral blood cells confirming the disorder. So, she had CD59 deficiency. The patient’s episodes were managed with plasma exchanges, corticosteroids, Cyclophosphamide, and Mycophenolate Mofetil which induced and maintained remission.

**Conclusion:**

CD59 deficiency can be presented with various clinical features such as neurologic, hematologic, dermatologic, and rheumatologic problems including systemic lupus erythematosus.

## Background

CD59 glycoprotein, also known as MAC-inhibitory protein (MAC-IP) is one of the cell surface glycoproteins that restrain the membrane attack complex (MAC) formation by stopping C9 unfolding. Glycophosphatidylinositol is a molecule that binds to CD59 glycoprotein of the cell membrane. A somatic mutation of PIG-A on chromosome X causes dysfunctional anchoring of CD59 to the cell membrane. MAC deposition is the consequence of this mutation in affected cells [1–3]. The final result of MAC formation is cytotoxicity, endothelial destruction and neuronal degeneration. These all are caused by transmembrane pore formation that is made by complement components including C5b, C6, C7, and C8 [4]. CD59 is essential for the regulation of the final steps of the complement pathway. Various deficiencies in complement pathway could be presented with various forms of autoimmunities including systemic lupus erythematosus (SLE) and lupus like syndrome. Herein, we presented a girl with serial clinical features of repeated acute inflammatory polyradiculoneuropathy, angioedema, paresthesia, myelitis, and finally malar rash and autoantibodies with final diagnosis of SLE and CD59 deficiency syndrome.

## Case presentation

A 16-year-old girl presented with unilateral facial edema on the right side with ptosis and hyperesthesia of the whole body with limb preference and quadriplegia. The patient complained of severe neck pain as well as severe headache and was unable to move her neck and head. In physical examinations, the forces of proximal and distal muscles of upper and lower extremities were 0 (no muscle activation). The patient was the first child of a consanguineous marriage. Her family history was unremarkable except for repeated urticaria in one of the patient’s uncle.

In the patient’s past medical history, she was admitted twice into the pediatric intensive care unit (PICU) at 15 and 30 months of age, because of progressive weakness, firstly in the lower limbs, and then in the upper extremities, followed by ptosis and drowsiness; with both episodes occurring after gastroenteritis. The patient was diagnosed with Guillain-Barré syndrome and during both admissions into PICU was treated with IVIG (intravenous immunoglobulin) and Methylprednisolone. During the second hospitalization, the patient developed fever, severe weakness, ptosis, and drowsiness, lasting for about a week, during which the patient was examined more thoroughly. One of these studies was EMG-NCV (electromyogram-nerve conduction velocity), which was reported as “severe demyelinating peripheral neuropathy”. Brain MRI reported “small T1 hypo, T2 hyper signal intensities in both middle cerebellar peduncles with extension in the cerebellar white matter on the right side”.

**Fig. 1:**
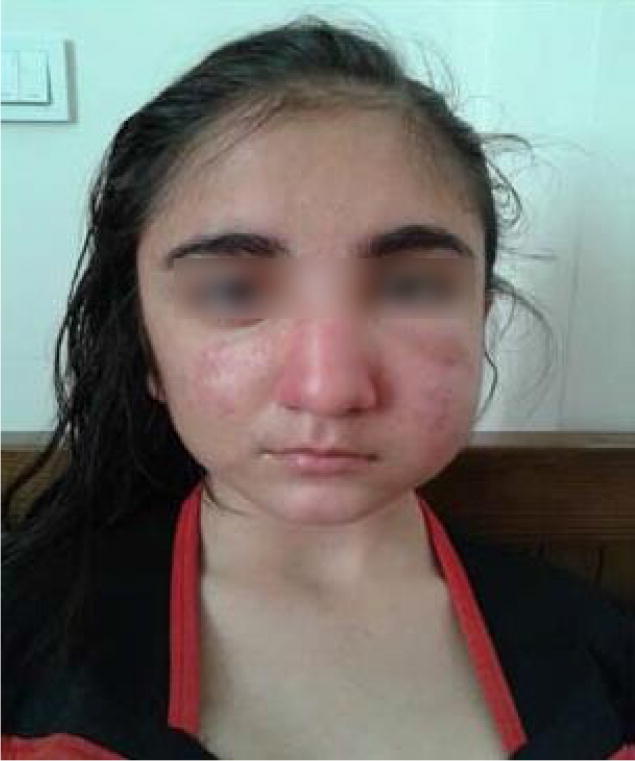
The adolescent girl with an attack of face angioedema

In laboratory studies serum lactate and ammonia, thyroid function tests, muscle enzymes, and autoantibodies specific to lupus were in normal ranges. During the second hospitalization, LDH (lactate dehydrogenase) was 856 Iu/l (normal < 480) and the patient’s aldolase level increased. A technetium-99 m brain SPECT (Single Photon Emission Computed Tomography) was also performed for her and mild hypoperfusion in the left frontal cortex was reported. After her general condition improved and the reversal of the patient’s deep tendon reflexes, she was discharged with a probable diagnosis of Miller Fischer syndrome and it was recommended that she continues her treatment with Prednisolone. This treatment continued until the age of 7, in conjunction with physical therapy and occupational therapy, due to the persistence of paresthesia and muscle weakness of the lower limbs. Gastrocnemius muscle biopsy was performed and the identical pathological diagnosis was reported by two different medical centers as stated below: (1) muscular atrophy, progressive spinal infantile type (Werding-Hoffman disease) (2) Skeletal muscle tissue with group (neurogenic) atrophy and chronic inflammatory demyelinating polyradiculoneuropathy. All treatment was discontinued after the age of 7 and the patient had no symptoms until 12 years of age, except for a few mild attacks of urticaria and periorbital edema, which were resolved rapidly by antihistamines such as Cetirizine and Hydroxyzine. During this period (7 to 12 years of age), she was also treated with growth hormone because of short stature. At the age of 13, she developed an attack of neck, chest and left upper extremity hyperesthesia, with unilateral facial edema (Fig. 1). These symptoms were preceded by an upper respiratory infection and fever, which again resulted in hospitalization. Symptoms decreased slightly with Methylprednisolone and Cetirizine. The brain MRI was repeated and was reported as “T2-FLAIR bright areas in left posterior parietal periventricular white matter as well as the left temporal area”. Her signs and symptoms were reduced after the attack but did not disappear completely. Various studies which included porphyria, were performed during the hospitalization to make the correct diagnosis; however the porphyria genetic test was negative. Due to repeated angioedema attacks, the possibility of allergic reaction was suggested, therefore serum specific IgE against a complete panel of allergens was measured, but there were no significant reactions to any allergens. Echocardiography and chest x-ray were normal. There were no pathologic findings in the abdominal and pelvic ultrasonography, except a 32 mm diameter cyst in the left ovary. Due to the weakness of the extremities, EMG-NCV was performed for her, which was normal. Laboratory data revealed no abnormal findings (Table 1). The patient was evaluated for the possibility of systemic lupus erythematosus as well as anti-phospholipid antibody syndrome with no positive findings. Furthermore, C1- Inhibitor level was normal. The paraclinical evaluation for Behçet’s disease was performed with an assessment of HLA-B5 and HLA-B51 as well as a Pathergy test, all of which were negative. The patient was also examined for the probability of primary immunodeficiency disorders by measurement of immunoglobulin levels and flow cytometry (CD3, CD4, CD8, CD16, CD19, CD20, CD56), all of which were reported within the normal range. The probability of Mycobacterium tuberculosis infection was also ruled out by a negative PPD test. The CSF fluid analysis for oligoclonal bands, A-NMO, Anti-MOG (myelin oligodendrocyte glycoprotein) antibody, protein, and glucose level, cell counts, as well as smear and culture were normal. In urine analysis, the only pathological finding was the presence of urobilinogen. MRI of the brain and spinal cord was reported as: “demyelinating lesions in occiput and mild inflammation within the nerve roots, and the spinal cord was normal”. With the improvement of angioedema, fever, and some improvements of the limbs weakness, and prior to the genetic test, she was treated in accordance with the probable diagnosis of complement deficiency and porphyria with Cetirizine, Prednisolone and Hydroxychloroquine. The patient was informed about the contraindication of use of ACE-inhibitors (Angiotensin-converting enzyme), NSAIDs and Aspirin and was discharged.

After 3 months, the patient was admitted again, because of angioedema, paresthesia and a facial rash similar to the previous admission. Symptoms had started after an episode of fever, cough, and pharyngitis. A urine analysis was performed and the results demonstrated the presence of blood with red blood cells/HPF and the result of the urine culture was negative. The patient was treated again with Methylprednisolone and fresh frozen plasma (FFP) due to a possible diagnosis of complement inhibitor deficiency. Subsequently, the patient’s clinical symptoms were alleviated and she was discharged from the hospital.

**Table 1:** Laboratory results in the CD59-deficient patient at 30-months-old and during the observation period

Test	At 30 months	At 12 years	At 16 years
CBC	Normal	Normal	Hb: 11.6 mg/dl, WBC: 4100/mm^3^, Absolute lymphocyte count: 890/mm^3^
ESR (mm/h)	2	10	23
CRP	1+	Negative	Negative
LDH (normal range < 480)	856	510	690
Uric acid (normal range)	–	3.4(2.4–5.7)	4.3(3.2–6.4)
CPK(normal range 24–229)	35	83	75
Aldolase (normal range 2–8)	15	6.7	5.6
ALT(normal range < 46)	30	20	15
AST(normal range < 31)	15	11	36
Serum electrolytes	Normal	Normal	Normal
FANA	Neg	1/20	1.8 (Neg < 1)
C3(normal range 90–180)	120	133	135
C4(normal range 10–40)	17	28.7	27
CH50(normal range 70–150)	138	125	131
Anti-ds DNA	0.9 (neg < 0.9)	1.5 (neg < 100)	Neg
Lupus anti-coagulant antibody	Neg	Neg	Neg
Anti-cardiolipin antibody (neg < 12)	2.1	1.7	Neg
Anti-B2 glycoprotein (neg < 10)	Neg	1.2	Neg
C1-inhibitor	–	0.355(Neg)	Neg
PT, PTT, INR	Normal	Normal	Normal
Lipid profile	–	–	Normal
Total protein	–	–	6.2(nl 5.7–8)
Albumin	–	–	4.3
Anti-SM antibody(IgG)	0.2(neg < 0.9)	Neg	Neg
Serum IgG	–	–	2047
Serum IgM/IgA/IgE	–	–	Normal
Urine analysis	Normal	Blood 1+ RBC: 8–10	Protein 1+
Protein (mg Urine 24 h)	–	–	2048 and 468
CSF analysis:			
Protein	Normal	9	72
Glucose	Normal	57	66
WBC	Normal	0	5
RBC	Normal	0	20
A-NMO	Normal	–	–
Anti-MOG	Normal	–	–
Oligoclonal band	Normal	–	–
HLA-B5/B51	–	Neg	–
Flow cytometry: CD3, CD4, CD8, CD16, CD19, CD20, CD56	–	Normal	–

Five months later, the patient again demonstrated similar symptoms such as fever, vertigo, abdominal pain, and vomiting and was treated with FFP, Methylprednisolone, and Ranitidine. She was subsequently discharged from the hospital with some improvement.

Regarding the relapsing symptoms and response to FFP, with a possible diagnosis of complement inhibitor deficiency, the patient was treated with FFP every month, which significantly improved the symptoms such as limbs weakness and paresthesia and the angioedema attacks did not reoccur either.

**Fig. 2:**
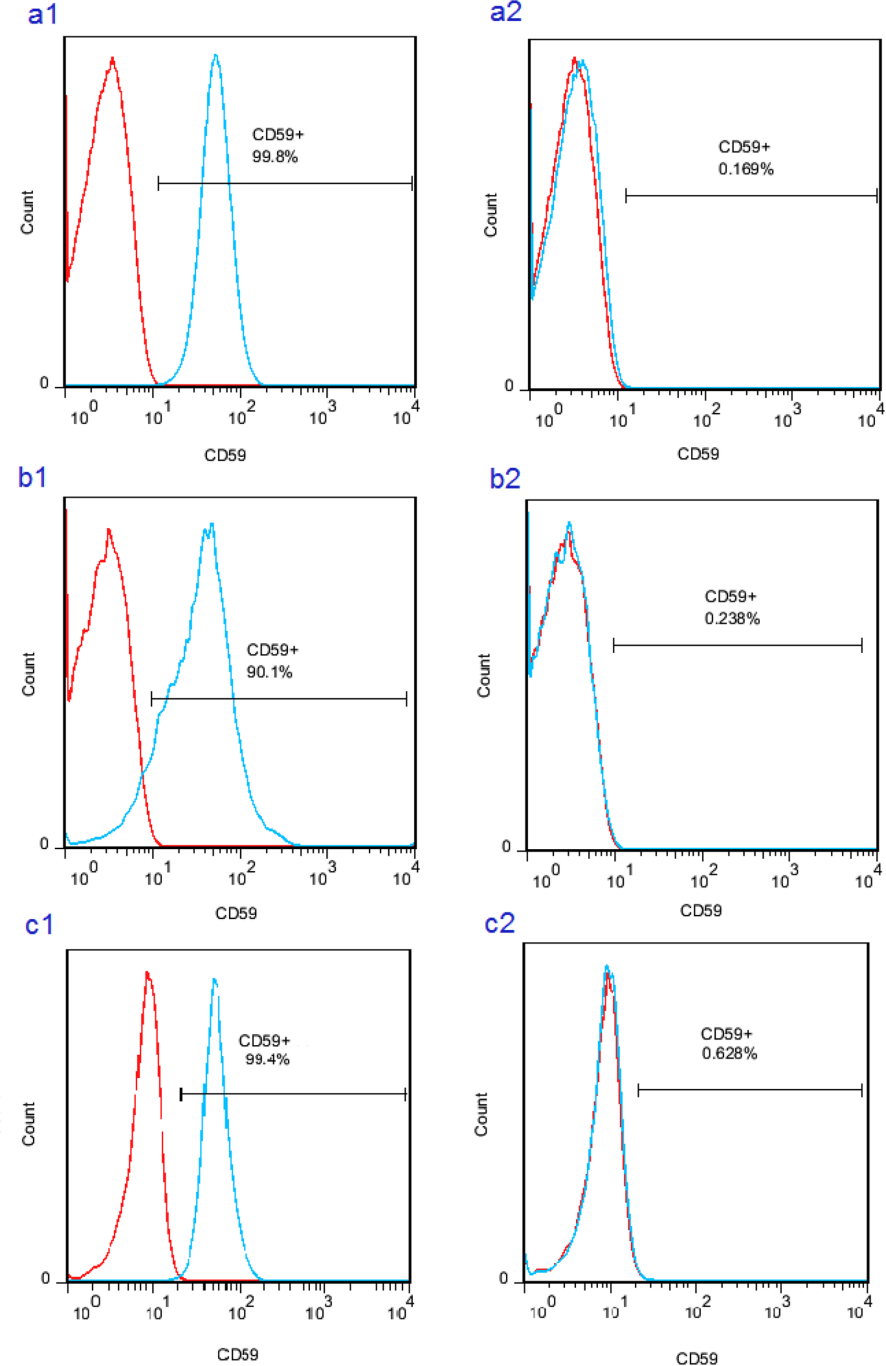
Flow cytometric analysis of CD59 expression in healthy control (1) and the patient (2) on the surface of RBCs (**a**), lymphocytes (**b**) and granulocytes (**c**)

Since no definite diagnosis was still made for the patient, and also the patient’s paresthesia had not improved completely, a whole-exome sequencing with Next-Generation Sequencing (NGS) method was performed for her. NGS reported a homozygous mutation in the CD59 gene (c.85T>G) that could explain the patient’s symptoms. This diagnosis was confirmed by genetic screening of the patient’s parents using the Sanger sequencing technique. Both parents had a heterozygous gene, which confirmed the autosomal inheritance of the disease. CD59 expression on the surface of peripheral blood lymphocytes, granulocytes and RBCs was also evaluated (Fig. 2), and the results demonstrated that CD59 was expressed on the cell surfaces of less than one percent of all studied cell populations. These results confirmed the diagnosis of CD59 deficiency.

Afterward, the patient, except for slight paresthesia in the left upper and lower limbs, had no problem until 16 years of age. At this time she was admitted because of severe neck pain and headache accompanying unilateral facial edema, ptosis, generalized hyperesthesia and four limbs paralysis. The patient was reevaluated. Initially, due to headache and neck stiffness, meningitis was suspected so brain CT scan was performed and due to its normality, the cerebrospinal fluid puncture was performed. CSF analysis was as follows: glucose 66 mg/dl, protein 72 mg/dl, WBC 5, RBC 20. According to the patient’s previous records, Methylprednisolone 2 mg/kg/daily and FFP were administered. This treatment relieved facial edema, with no improvement in her paralysis and other symptoms. In paraclinical evaluation (Table 1), CBC showed lymphopenia and mild anemia (Hb = 11.6). C3, C4, and CH50 were in the normal range. CRP, Anti-ds DNA, Anti-Sm, Lupus anticoagulant, Anti-SSA, Anti-SSB, anticardiolipin Abs (IgG, IgM), β-2 glycoprotein Ab (Ig G, IgM) were negative. ANA titer was 1.8 (neg < 1). ESR was 23 mm/h. In the immunoglobulin assay, IgG was 2047 mg/dl and other immunoglobulins were normal. Proteinuria was reported in urine analysis so two 24-h urine collections were prescribed for the patient. The results of the first urine collection showed a 2048 mg of protein and the second result showed 468 mg of protein. Based on the electrophoresis of urine proteins and a consultation with a nephrologist, no kidney biopsy was performed. MRI of the brain and spinal cord was performed with and without intravenous contrast media with the following results: “Deep white matter of right cerebellum shows elongated local medium size brightness at T2W, FLAIR. Both parietal lobes show few thin UBO’s (unidentified bright objects including demyelinating process). The cervical cord showed elongated bright signal in the posterior column with central cord canal ectasia at the entire course of it without expansion. The lesion was not enhanced (in favor of demyelinating process)”.

EMG-NCV was also performed, which was reported as follows: “Old and chronic axonal polyneuropathy in lower limbs and there is no evidence of motor neuron disease and myopathy in limbs”.

Due to the patient’s non-responsiveness to Methylprednisolone (2 mg/kg/day) and FFP, the patient was administered 3 consecutive days of Methylprednisolone pulse (30 mg/kg/day) and 5 days of IVIG (1 g/kg/day), which did not improve the patient’s condition significantly. The patient underwent plasmapheresis for 8 consecutive days, which resulted in partial improvement in neck pain and improvement of neck movement. Legs’ movements improved but recovery was slow in the hands as the left hand was completely paralyzed and in the right hand, she was only able to move her fingertips. Due to the neurological symptoms, positive ANA, proteinuria, lymphopenia and appearance of malar rash, she was diagnosed with SLE and treated accordingly. Subsequently, she received Cyclophosphamide pulse, which improved the movement of the legs and neck, but the movement of the hands remained unchanged.

In accordance with the SLE diagnosis, she was treated with Hydroxychloroquine, Prednisolone, and monthly Cyclophosphamide. With a 6-month induction of remission, clinical and laboratory manifestations of SLE were controlled. Her gait and the strength of her lower limbs improved significantly. She returned to her daily usual activities with minimal impairment in her fine motor skills such as hand writing.

## Discussion

In the case presented, the patient’s symptoms began with progressive weakness in the lower limbs, which reoccurred many times in a similar manner for many years. As the result, the patient was diagnosed with Guillain-Barre syndrome by a pediatric neurologist. The patient was first treated with IVIG and Methylprednisolone pulses and subsequently with low-dose Prednisolone. The patient’s symptoms were controlled with these medications and the treatment was therefore discontinued. The same onset of our patient’s symptoms has also been reported in similar cases [5]. Furthermore, the demyelinating changes in our patient’s brain MRI were also observed in other previously reported cases [6].

Due to repeated attacks and inadequate response to treatment, whole-exome sequencing was performed to investigate possible unknown causes. The result was homozygous for CD59 deficiency for the patient and heterozygous for her parents. Our patient did not have paroxysmal nocturnal hemoglobinuria (PNH) and Coombs negative hemolysis in contrast with previously reported cases [4]. Demyelination, stroke and Guillain-Barre syndrome are neurological manifestations which are described in congenital CD59 gene mutations [7]. Recurrent cerebral infarctions, moyamoya syndrome, hemolytic uremic syndrome (HUS) like disease, atypical glomerulonephritis, and necrotic fingers are the other clinical features which have been reported in the literature [8]. However, in our patient’s last hospitalization, due to her failure to respond to her own previously and similarly administered treatments which led to quadriplegia accompanying severe headache and angioedema, the patient was re-evaluated. In the re-evaluation tests, ANA was positive, and proteinuria was in range of nephrotic syndrome. During hospitalization, she developed a malar rash, which with accompanying clinical symptoms and laboratory results was diagnosed as SLE in the presence of CD59 Deficiency.

Although previous reports had expressed the efficacy of Eculizumab in patients with CD59 deficiency and neurological symptoms [9], we did not have access to this medication for administration to our patient. Furthermore, our patient manifested clinical and laboratory symptoms of lupus (SLE) and lack of response to steroids and IVIG as well as renal involvement. Therefore, an eight-day course of plasmapheresis and subsequently a proper dose of intravenous pulse of Cyclophosphamide were administered. This treatment markedly improved the patient’s clinical symptoms. The induction of remission with Cyclophosphamide continued for the patient with monthly intravenous administration for six consecutive months. Furthermore, the patient was treated with appropriate doses of Hydroxychloroquine and Prednisolone. Subsequently, our patient’s treatment was continued with sequential therapy with Mycophenolate Mofetil. Physical therapy and occupational therapy were administered as adjunctive therapies.

## Data Availability

The datasets used and analysed during the current study are available from corresponding author on reasonable request.
